# GATA3 and markers of epithelial-mesenchymal transition predict long-term benefit from tamoxifen in ER-positive breast cancer

**DOI:** 10.1038/s41523-024-00688-6

**Published:** 2024-09-06

**Authors:** Josefine Sandström, Jens Bomanson, Gizeh Pérez-Tenorio, Carolin Jönsson, Bo Nordenskjöld, Tommy Fornander, Linda S. Lindström, Olle Stål

**Affiliations:** 1 Department of Biomedical and Clinical Sciences and Department of Oncology, 581 83 Linköping University, Linköping, Sweden; 2 Department of Oncology and Pathology, Karolinska Institute and University Hospital, Stockholm, Sweden; 3 Breast Center, Karolinska Comprehensive Cancer Center, Karolinska University Hospital, Stockholm, Sweden

**Keywords:** Breast cancer, Breast cancer

## Abstract

GATA binding protein 3 (GATA3) is essential for normal development of the mammary gland and associated with ER-positive breast cancer. Loss of GATA3 has been associated with epithelial-mesenchymal transition (EMT) in experimental studies. We investigated tumoral GATA3 in a cohort of postmenopausal patients with lymph-node negative breast cancer, randomized to adjuvant tamoxifen or control. Nuclear GATA3 expression was assessed with immunohistochemistry and GATA3 gene expression with Agilent microarrays. High GATA3 nuclear expression was associated with a lower rate of distant recurrence in ER-positive breast cancer (HR = 0.60, 95% CI 0.39–0.93). Low gene expression of GATA3 was associated with limited long-term benefit from adjuvant tamoxifen (interaction: *p* = 0.033). GATA3 gene expression was associated with the epithelial markers CDH1 (E-cadherin) and FOXA1, whereas negatively associated with several mesenchymal markers. Low expression of CDH1 was associated with marginal tamoxifen benefit (HR = 0.80 (0.43–1.49)), whereas patients with higher expression showed a significant benefit (HR = 0.33 (0.20–0.55), interaction: *p* = 0.029). In ER-positive breast cancer, diminished expression of GATA3 is associated with markers of EMT and poor long-term benefit from tamoxifen.

## Introduction

Breast cancer commonly arises in luminal cells of the mammary gland expressing the estrogen receptor (ER). The expression of ER in the tumor is a cornerstone for the selection of adjuvant treatment. Patients with ER-positive breast cancer receiving endocrine therapy have initially a good prognosis, but studies with long-term follow-up have shown that there is a continuous risk of late relapse of the disease for these patients^1–3^. The endocrine treatment lasts for five or even ten years and there is a need for better prediction of overtreatment as well as of development of treatment resistance and late relapse.

The range of target genes regulated by ER is dependent on the phosphorylation of ER at different sites and cofactors that bind to DNA in the proximity of ER-binding sites^4^. GATA binding protein 3 (GATA3) and forkhead box A1 (FOXA1) are two proteins forming a strong transcriptional network together with ER. This network is required for correct development of the mammary gland^5,6^. ER-positive tumors often express GATA3, but a low GATA3 expression has been associated with worse prognosis^7,8^. However, whether GATA3 is an independent prognostic factor, and whether it predicts the benefit from tamoxifen, has yet not been settled. The importance of phosphorylated ER (pER) for the efficacy of tamoxifen has been previously studied by others and by us^9–12^. The receptor is phosphorylated at different sites by intracellular signaling molecules including MAP-kinase (pERser118), S6K1 (pERser167) and, PKA and PAK1 (pERser305)^11–14^.

Loss of functional GATA3 can arise due to mutations in the gene, which have been found in 10–15% of ER-positive breast cancer^15^. Mutations seldom silence the GATA3 gene- or protein expression but could alter its function. Without functional GATA3, or perhaps with too much of a function, the transcriptional landscape will change. Experimental studies suggest that loss of GATA3 expression is associated with epithelial-mesenchymal transition (EMT), which in turn might facilitate dissemination of tumor cells and the establishment of metastases^16^. To the best of our knowledge, the relationship between GATA3 and EMT factors in human breast tumors has not been thoroughly investigated.

The functionality of GATA3 can be disrupted by gene mutations or could be reduced by decreased gene expression or increased protein degradation. Here, we investigate the tumoral expression of GATA3 in a cohort of postmenopausal patients with lymph-node negative breast cancer, randomized to adjuvant tamoxifen or no systemic treatment. With gene expression data from the same cohort, we analyze several genes involved in EMT and cell adhesion in relation to GATA3 in ER-positive breast cancer. Furthermore, we examine the long-term prognosis and benefit from adjuvant tamoxifen in association to GATA3 and related factors.

## Methods

### Patient cohort

In a trial conducted by the Stockholm Breast Cancer Study Group, postmenopausal breast cancer patients with a negative lymph node status and tumor size not exceeding three cm were randomized to adjuvant tamoxifen, 40 mg daily for two years or no tamoxifen, the Stockholm tamoxifen trial STO-3^17^. The trial recruited patients regardless of ER status. Patient entry to the study was from November 1976 to May 1990. In 1983, tamoxifen-treated recurrence-free patients were randomized, if consenting, to three more years of tamoxifen or no further treatment with tamoxifen. The follow-up period lasted at most to 30 years with a median of 22.6 years of follow-up.

**Fig. 1:**
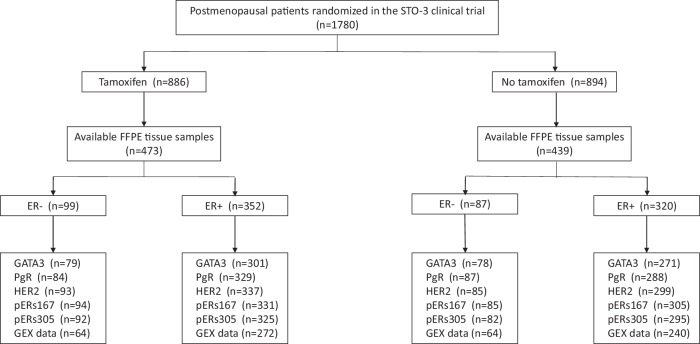
Flowchart of included patients. Formalin-fixed paraffin-embedded (FFPE), Gene expression (GEX).

Figure 1 shows a flowchart of the study cohort. For 912 of the 1780 patients in the trial, formalin-fixed paraffin-embedded tumor tissue was available and used to construct tissue micro arrays (TMAs) with three tissue cores from each tumor. Previously, data for samples on the TMA were compared with data of the original cohort of 1780 patients^9^. The results showed no bias with respect to tumor size, ER status, or treatment arm. The STO‐3 trial was approved by the ethics committee at Karolinska Institutet (KI) in Stockholm, Sweden, and participants provided oral consent (KI 76–51). Further, the ethics committee at KI approved retrospective studies on archived tumor tissue for the present cohort, with the purpose to evaluate prognostic and treatment predicting factors (KI 97–451 with amendments 030201 and 2017 2066-32). Further need for patient consent was waived by the ethics committee.

### Biomarkers previously investigated in this cohort

Immunohistochemistry of ER, progesterone receptor (PgR) and HER2 was previously assessed^18,19^. Further, for ER phosphorylated at two different sites (pER), pERs167 and pERs305, data were available^9,10^. The antibodies used for ER, PgR and HER2 were, respectively, the CONFIRM™ mouse anti-ER antibody (clone 6F11) and the CONFIRM™ mouse anti-PR antibody (clone 16) from Ventana Medical Systems, and the DAKO AO0485 polyclonal rabbit antibody according to the guidelines provided by the manufacturer. The antibodies for pER were a rabbit polyclonal pERα^ser305^ primary antibody (Bethyl Laboratories, Montgomery, TX) and anti-pERs167 from Cell Signaling Technologies (Danvers, MA).

### GATA3 protein staining

The GATA3 protein was investigated with immunohistochemistry on TMA sections. Several studies comparing antibody sensitivity revealed the L50-823 as the most sensitive GATA3 antibody compared to another commonly used antibody, HG3-31, reviewed in Kandalaft et al.^20^. The PT-link station was used for deparaffinization and antigen retrieval in a low-pH buffer (K800521-2, EnVision FLEX Target Retrieval Solution, DakoCytomation, Glostrup, Denmark), starting at 65 °C, gradually increasing and ending at 96 °C for 20 min and cooled down to 65 °C. Inactivation of endogenous peroxidase in 3% hydrogen peroxide in water was followed by blocking in serum-free protein block for 10 min (DPB-125, Spring Bioscience, Freemont, CA). TMA sections were incubated in a moisturized chamber at 4 °C during 24 h with the anti-GATA3 mouse monoclonal antibody diluted 1:500 (L50-823, Merck KGaA, Darmstadt, Germany). Secondary anti-mouse antibody (K4000, DakoCytomation Envision+ HRP system, Agilent Technologies, CA) was applied for 30 min at room temperature and protein staining was developed with 3,3’-diaminobenzidine chromogen and substrate buffer, dilution 1:50, for 8 min (K3467, DakoCytomation, Agilent Technologies, CA) and counterstained with hematoxylin for 1 min. All washing steps were in phosphate buffered saline including 0.5% bovine serum albumin. The tissue was dehydrated, and cover glass was mounted with Pertex (00871, Histolab, Askim, Sweden). Slides were visualized using the Aperio CS2 brightfield digital scanner at ×400 magnification and analyzed with the ImageScope software (Leica biosystems, Buffalo Grove, IL).

### GATA3 protein grading

Of the 912 tumors, 749 were successfully stained and graded for GATA3 protein expression. Nuclear staining intensity was graded in three steps: negative (0), weak (1) and strong (2). Frequency of positive tumor nuclei were scored as follows; 0% (0), 1–10% (1), 11–50% (2), 51–89% (3) and ≥90% (4). For statistical analysis, the nuclear staining was divided in low and high, with a cut-off defining the group with high expression as strong nuclear staining in >50% of tumor cells. Primary grading was performed by two independent observers (J.S. and J.B.), blinded to clinical data, and secondary grading was performed jointly by the two observers to reach consensus.

### Gene expression analysis

Messenger RNA was extracted from FFPE breast tumor tissue and 652 samples were available for microarray gene-expression analysis using custom-designed arrays, containing 32.1 K probes, detecting about 21.5 K unique genes (Agilent Technologies, CA)^21^. The Prediction Analysis of Microarray 50 (PAM50) intrinsic subtype analysis classifier was used as described by Parker et al.^22^.

We selected a number of genes encoding proteins known to interact with GATA3 or EMT. FOXA1 is a strong transcriptional partner of GATA3 and NOTCH3 might upregulate GATA3. Several factors related to EMT are known from the literature. Among them, we selected E-cadherin (CDH1), an epithelial marker frequently lost in EMT. On the other hand, N-cadherin (CDH2) and the intermediate filament protein vimentin (VIM) are expressed in mesenchymal cells. In addition, we selected alpha-smooth muscle actin (ACTA2) that is associated with the TGF-β pathway, which in turn can drive the EMT process. Finally, the EMT-activating transcription factors Snail (SNAI1) and Twist-related protein 1 (TWIST1) were included in the list of genes analyzed.

Expression levels of GATA3 and other genes were analyzed by tertiles (T1-T3) in the association analyses. In the survival analyses, the lowest tertile (T1) was used as cut-off for GATA3, CDH1 and FOXA1, as the low expression was expected to stand out from the group as more aggressive. For the EMT-related genes, the cut-off was set at the highest tertile (T3), as a high expression of these genes was expected to stand out from the remaining group as more aggressive. In the multiple regression analysis, the gene-expression levels were analyzed as continuous variables.

### Statistics

Statistical analyses were performed using Statistica 14 (TIBCO Software Inc.). For comparisons of GATA3 protein expression with other characteristics, the Pearson χ2 test was applied for 2 × 2 tables. For associations of GATA3 gene expression in three categories with other factors, the Spearman rank order correlation was applied. Distribution of GATA3 in the PAM50 molecular signatures was compared with the Kruskal–Wallis test. Multiple linear regression analysis was used to investigate how each mesenchymal marker could be predicted by GATA3, FOXA1 and NOTCH3. Distant recurrence-free interval (DRFI) time distributions were compared, and plots were drawn with the Kaplan–Meier method, visualizing time from randomization to first event of distant metastasis. Hazard ratios (HRs) of distant metastasis were estimated using the Cox proportional hazards model. Cox models were furthermore applied in multivariable analysis and in interaction analysis exploring the expression of genes as potential predictive factors for tamoxifen treatment benefit. A *p*-value of less than 0.05 was considered significant.

## Results

**Fig. 2:**
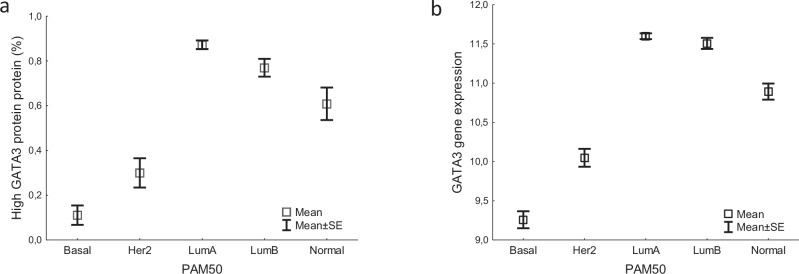
Among breast cancer subtypes, the Luminal A subtype shows the highest levels of GATA3. The fraction of tumors with high GATA3 protein expression (**a**) and gene expression levels (**b**) in relation to the PAM50 subtypes. Kruskal–Wallis H-test, *p* < 0.0001 for both comparisons.

Overall, 70% of the tumors exhibited high nuclear GATA3 expression and GATA3 was strongly associated with ER status. Among ER-positive tumors, high nuclear and mRNA GATA3 expression, was seen in 84% and 41% of the cases, respectively. Corresponding numbers for ER-negative tumors were 19% and 5%, respectively (*p* < 0.0001 for both). Accordingly, the PAM50 molecular subtypes differed in relation to GATA3, for both nuclear and mRNA expression, with Luminal A showing the highest levels and the Basal subtype the lowest (Fig. 2). Therefore, we restricted the analyses in the following to patients with ER-positive breast cancer and the tertiles for GATA3 were from now on based on this subgroup.

### Associations of GATA3 with clinicopathological variables and pER

Nuclear GATA3 was more frequently expressed at high levels in small and PgR-positive tumors (Table 1). HER2 positivity was significantly associated with low GATA3 gene expression levels and a similar trend was seen for GATA3 protein expression (Tables 1 and 2). Both protein and gene expression levels of GATA3 were associated with ER phosphorylated at serine-305 (pERs305), whereas the association of GATA3 with pERs167 was less clear.

**Table 1:** GATA3 nuclear protein expression in relation to clinicopathological variables, ER-phosphorylation and GATA3 mRNA by tertiles (T) in ER-positive tumors

		GATA3 nuclear protein		
		Low	High	
		*n* (%)	*n* (%)	*p*-value
Tamoxifen	Untreated	51 (19)	220 (81)	
	Treated	39 (13)	262 (87)	0.055
Tumor size	<20 mm	57 (12)	405 (88)	
	≥20 mm	33 (33)	67 (67)	**<0.00001 (−)**
PgR	<10%	42 (23)	140 (77)	
	≥10%	42 (12)	298 (88)	**0.0015**
HER2	Negative	76 (15)	423 (85)	
	Positive	9 (26)	25 (74)	0.083
pERs167 nuclear	Negative	77 (18)	354 (82)	
	Positive	13 (10)	121 (90)	**0.024**
pERs305 nuclear	Negative	70 (21)	269 (79)	
	Positive	19 (9)	195 (91)	**0.00024**
GATA3 mRNA	T1	36 (24)	112 (76)	
	T2	23 (16)	124 (84)	
	T3	12 (8)	136 (92)	**0.00013**^**a**^

*P*-values for significant associations in bold. A significant negative association is indicated with a minus sign.

^a^*P*-value from Spearman rank order correlation.

**Table 2:** GATA3 gene expression in relation to clinicopathological variables and ER-phosphorylation in ER-positive tumors

		GATA3 gene expression by tertiles (T)			
		T1	T2	T3	
		*n* (%)	*n* (%)	*n* (%)	*p*-value^a^
Tamoxifen	Untreated	83 (35)	75 (31)	82 (34)	
	Treated	88 (32)	96 (35)	88 (32)	0.95
Tumor size	≤20 mm	137 (33)	139 (34)	138 (33)	
	≥20 mm	31 (34)	30 (33)	31 (34)	0.98
PgR	<10%	56 (37)	38 (25)	57 (38)	
	≥10%	104 (33)	115 (36)	98 (31)	0.76
HER2	Negative	146 (32)	151 (34)	153 (34)	
	Positive	18 (69)	4 (15)	4 (15)	**0.00076 (−)**
pERs167 nuclear	Negative	132 (35)	121 (32)	121 (32)	
	Positive	33 (29)	40 (35)	40 (35)	0.30
pERs305 nuclear	Negative	117 (39)	99 (33)	83 (28)	
	Positive	44 (25)	59 (33)	76 (42)	**0.00014**

*P*-values for significant associations in bold. A significant negative association is indicated with a minus sign.

^a^*P*-value from Spearman rank order correlation.

There was a significant correlation between GATA3 mRNA and protein levels (Table 1), but this relationship showed differences dependent on pER. For tumors with a positive status of pERs167, nuclear GATA3 was frequently highly expressed also at low GATA3 mRNA levels, resulting in no correlation between gene and protein expression levels.

**Fig. 3:**
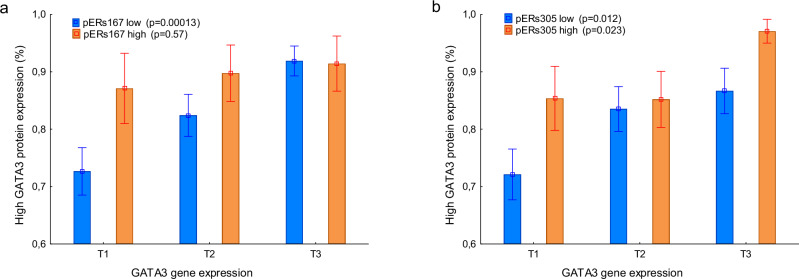
The correlation between GATA3 gene and protein expression depending on pER. The relationship between GATA3 gene and protein expression by the status of pERs167 (**a**) and pERs305 (**b**). The *p*-values refer to Spearman rank order correlation and error bars refer to standard error of the mean. Tertile (T).

On the other hand, the subgroup of pERs167- negative tumors showed a significant gene to protein correlation (Fig. 3). In contrast, the status of pERs305 did not markedly affect the correlation between gene and protein expression levels.

### Associations of GATA3 with epithelial and mesenchymal biomarkers

Based on previous experimental research showing that the loss of GATA3 may contribute to EMT, we next analyzed the relationship between GATA3 and the expression of several genes associated with this process. Comparing gene-expression levels, GATA3 was negatively correlated with all the mesenchymal biomarkers investigated, including ACTA2, CDH2, SNAI1, TWIST1 and VIM (Table 3). For GATA3 protein levels, the same was true for three of the biomarkers. Furthermore, the gene expression of GATA3 was positively associated with CDH1, FOXA1, and NOTCH3.

**Table 3:** GATA3 in association with epithelial and mesenchymal biomarkers in ER-positive breast cancer

	GATA3 mRNA *n* (%)				GATA3 protein *n* (%)		
	T1	T2	T3	*p*	Low	High	*p*
CDH1 (E-cadherin)				**0.00020**			0.10
T1	79 (39)	67 (32)	43 (24)		22 (15)	129 (85)	
T2	57 (33)	60 (35)	54 (32)		18 (12)	132 (88)	
T3	44 (27)	44 (27)	73 (45)		31 (22)	111 (78)	
FOXA1				**<0.00001**			0.43
T1	71 (54)	43 (33)	18 (14)		20 (18)	90 (82)	
T2	61 (33)	64 (34)	62 (33)		27 (16)	141 (84)	
T3	39 (20)	64 (33)	90 (47)		24 (15)	141 (85)	
NOTCH3				**0.00078**			0.83
T1	72 (39)	59 (32)	54 (29)		27 (16)	138 (84)	
T2	63 (36)	64 (37)	47 (27)		24 (16)	124 (84)	
T3	36 (24)	48 (31)	69 (45)		20 (15)	110 (85)	
ACTA2 (α-sma)				**0.00025 (−)**			**0.0010 (−)**
T1	38 (23)	56 (34)	71 (43)		15 (10)	129 (90)	
T2	66 (38)	52 (30)	54 (31)		20 (13)	132 (87)	
T3	67 (38)	63 (36)	45 (26)		36 (24)	111 (76)	
CDH2 (N-cadherin)				**<0.00001 (−)**			**0.023 (−)**
T1	30 (18)	54 (32)	84 (50)		18 (12)	130 (88)	
T2	55 (32)	55 (32)	61 (36)		21 (14)	128 (86)	
T3	86 (50)	62 (36)	25 (14)		32 (22)	114 (78)	
SNAI1 (Snail)				**<0.00001 (−)**			**0.0042 (−)**
T1	42 (23)	57 (32)	81 (45)		16 (10)	142 (90)	
T2	61 (35)	63 (36)	50 (29)		25 (17)	126 (83)	
T3	68 (43)	51 (32)	39 (25)		30 (22)	104 (78)	
TWIST1				**<0.00001 (−)**			0.98
T1	27 (16)	55 (34)	82 (50)		22 (15)	123 (85)	
T2	62 (35)	63 (36)	50 (29)		26 (18)	122 (82)	
T3	82 (47)	51 (31)	38 (22)		23 (15)	127 (85)	
VIM (Vimentin)				**0.00010 (−)**			0.74
T1	44 (25)	50 (29)	79 (46)		21 (13)	135 (87)	
T2	60 (35)	65 (38)	47 (27)		30 (20)	119 (80)	
T3	67 (40)	56 (34)	44 (26)		20 (14)	118 (86)	

*P*-values for significant associations in bold. A significant negative association is indicated with a minus sign.

Since the transcription factors GATA3 and FOXA1 have been suggested to inhibit EMT, and NOTCH3 could be a contributing factor, we performed multiple regression analysis to investigate these factors as independent predictors of CDH1 and the mesenchymal markers, respectively (Table 4). GATA3 turned out to be the variable that most consistently correlated with the markers investigated.

**Table 4:** Multiple regression of the EMT markers, respectively, based on GATA3, FOXA1 and NOTCH3 gene expression (continuous variables)

	mRNA		
mRNA	GATA3	FOXA1	NOTCH3
CDH1 (E-cadherin)	***p*** = **0.00001**	ns	ns
ACTA2 (α-sma)	***p*** = **0.000002 (−)**	***p*** = **0.00026 (−)**	***p*** < **0.000001**
CDH2 (N-cadherin)	***p*** < **0.000001 (−)**	ns	***p*** < **0.000001 (−)**
SNAI1 (Snail)	***p*** = **0.000014 (−)**	ns	ns
TWIST1	***p*** = **0.0011 (−)**	***p*** = **0.00045 (−)**	***p*** = **0.0013 (−)**
VIM (Vimentin)	***p*** < **0.000001 (−)**	***p*** = **0.0029 (−)**	***p*** < **0.000001**

*P*-values for significant associations in bold. A significant negative correlation is indicated with a minus sign.

### Distant recurrence-free interval in relation to GATA3

**Fig. 4:**
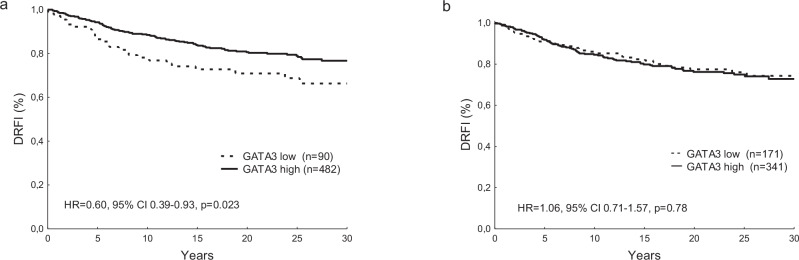
A high nuclear GATA3 expression predicts a favorable prognosis. Distant recurrence-free interval (DRFI) in relation to GATA 3 nuclear expression (**a**) and GATA3 gene expression (**b**). HR Hazard ratio, CI Confidence interval.

Patients with high tumoral nuclear GATA3 expression had a longer DRFI than those with low GATA3 levels (HR = 0.60, 95% CI 0.39–0.93, *p* = 0.023, Fig. 4a). When adjusting for treatment and other tumor characteristics, including tumor size, PgR and HER2, the statistical significance for GATA3 was lost (HR = 0.81, 95% CI 0.50–1.31, *p* = 0.39), whereas tumor size (>20 mm vs ≤20 mm; HR = 2.16, 95% CI 1.39–3.36, *p* = 0.00067) and tamoxifen (HR = 0.45, 95% CI 0.30–0.67, *p* < 0.0001) were significant. We did not find a significant association of GATA3 gene expression levels with DRFI (T2-T3 vs T1; HR = 1.06, 95% CI 0.71–1.57, *p* = 0.78, Fig. 4b).

### The benefit from adjuvant tamoxifen in relation to GATA3 and related factors

**Fig. 5:**
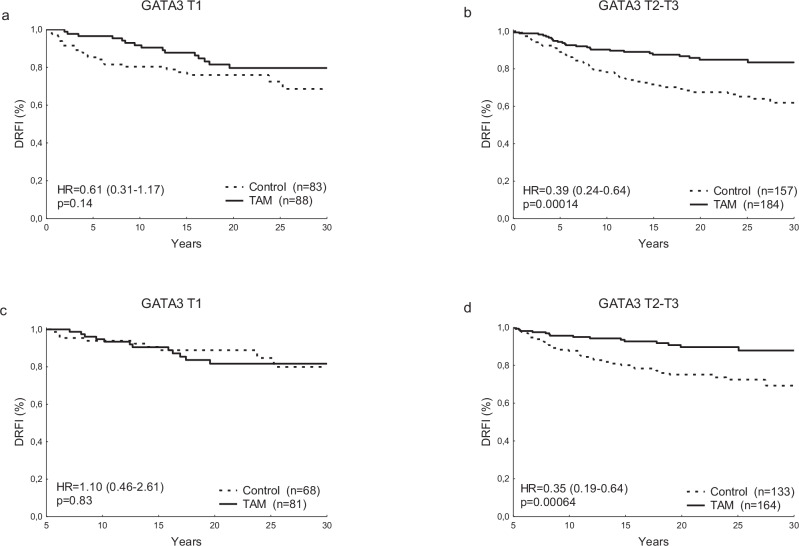
GATA3 gene expression as a predictive factor of tamoxifen benefit. DRFI for the tamoxifen and control groups in patients with low (**a**) and medium/high (**b**) GATA3 levels. DRFI for the tamoxifen and control groups, in patients alive and free of distant recurrence after five years, with low (**c**) and medium/high (**d**) GATA3. Hazard ratio (HR), tamoxifen (TAM), tertile (T).

Given that GATA3 and ER interact, the benefit from tamoxifen could potentially be dependent on expression levels of GATA3. Whereas patients with ER-positive tumors with intermediate to high GATA3 mRNA levels showed significant benefit from tamoxifen (HR = 0.39 (0.24–0.64), *p* = 0.00014), those with levels in the bottom tertile did not as evidently benefit (HR = 0.61 (0.31–1.17), *p* = 0.14) (Fig. 5). The difference was more pronounced when considering the long-term prognosis. For patients still alive and without a distant recurrence after five years, there was no further benefit from tamoxifen in the group with low GATA3 (HR = 1.10 (0.46–2.61), *p* = 0.83), in contrast to the group with higher levels (HR = 0.35 (0.19–0.64), *p* = 0.00064). A test for interaction between GATA3 and tamoxifen for this period was significant (*p* = 0.033). The efficacy of tamoxifen did not significantly differ for patients with low or high tumoral nuclear GATA3 protein expression (HR = 0.53 (0.23–1.23) and HR = 0.52 (0.34–0.79), respectively), however the number of patients in the former group was small.

**Fig. 6:**
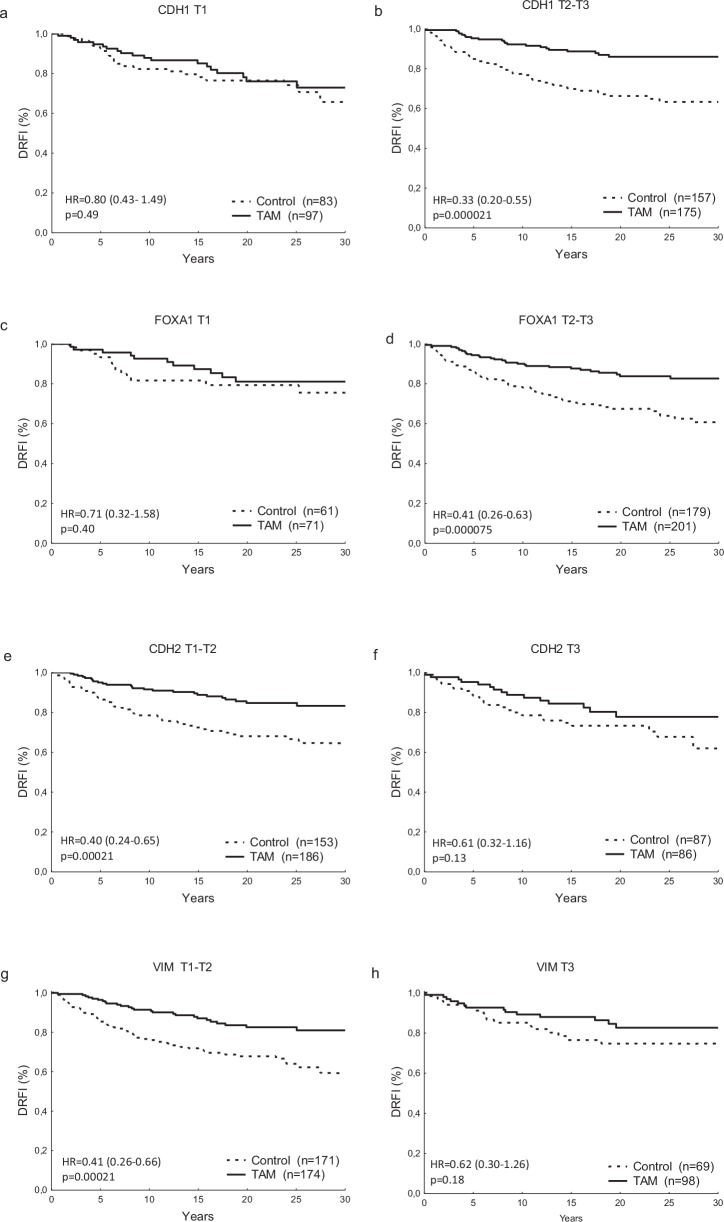
The gene expression level of CDH1, which encodes E-cadherin, predicts the benefit of tamoxifen. DRFI for the tamoxifen and controls groups in patients with low (**a**) and medium/high tumor CDH1 (**b**), in patients with low (**c**) and medium/high tumor FOXA1 (**d**), in patients with low (**e**) and medium/high tumor CDH2 (**f**), in patients with low (**g**) and medium/high tumor VIM (**h**). Hazard ratio (HR), tamoxifen (TAM), tertile (T).

The GATA3-associated genes CDH1 and FOXA1 in addition tended to predict the efficacy of tamoxifen. Patients with CDH1 levels in the bottom tertile experienced no evident benefit from tamoxifen (HR = 0.80 (0.43–1.49), *p* = 0.49), whereas those with higher levels did (HR = 0.33 (0.20–0.55), *p* = 0.000021), and the interaction was significant (*p* = 0.029, Fig. 6a, b). Although a similar interaction between FOXA1 and tamoxifen did not reach statistical significance (*p* = 0.22), the benefit from the treatment was more evident in the group with higher levels as compared with the group with low levels (HR = 0.41 (0.26–0.63), *p* = 0.000075) and (HR = 0.71 (0.32–1.58), *p* = 0.40), respectively, (Fig. 6c, d). Moreover, the EMT biomarkers CDH2 and VIM tended to predict less tamoxifen benefit when expressed at higher levels as compared to low levels (Fig. 6E–H). For the remaining EMT markers, the benefit from tamoxifen was similar comparing groups with low/intermediate versus high levels (tests for interaction, all *p* > 0.7).

## Discussion

GATA3, known as a marker used to identify mammary or urothelial origin of metastases from unknown primary tumors, is widely expressed in breast tumors and has been suggested as a potential prognostic and/or treatment predictive biomarker^23,24^. A quantitative decrease or functional loss of GATA3 seems to interfere with the characteristics of the tumor^25^. In line with previously published data, we see a distinct nuclear expression of GATA3 in more than 80% of ER-positive tumors as compared to in only about 20% of the ER-negative tumors^7,26,27^. GATA3 positivity was largely associated with a PAM50 Luminal subtype, supporting its functional role as co-regulator of the ER and its association with differentiation.

The ER protein is phosphorylated at distinct sites when regulated. The tight connection between ER and GATA3 suggests that the two proteins may regulate each other. We found a significant association between pERs305 and high GATA3 expression. We suggest that this might be related to PKA, which, besides its involvement in ERs305 phosphorylation^11^, has been shown to interact with GATA3^28^. Overall, there was a strong association of GATA3 gene expression with GATA3 protein expression. Interestingly, in tumors phosphorylated at ERs167, GATA3 gene- and protein expression did not correlate. Hypothetically, a stabilization of the GATA3 protein could be affected by intracellular signaling proteins associated with ER phosphorylation, supported by the finding that MAPK controls GATA3 protein stability by a post-transcriptional mechanism^29^. Furthermore, S6K1, that phosphorylates ER at serine 167, interacts with ER in a positive feedback loop also involving GATA3^30^.

We found low GATA3 protein levels to be significantly associated with increased risk of distant recurrence when compared to high GATA3 protein levels in the group of patients with ER-positive tumors. Mehra et al. reported already in 2005 that detection of GATA3 with immunohistochemistry could predict outcome of breast cancer, also when adjusting for other prognostic factors^8^. Several studies have found similar results^7,27,31^, although the independent prognostic value was absent in one of the studies when the analysis was restricted to ER-positive breast cancer^31^, similar to our results. Moreover, GATA3 was not independently prognostic in a huge study comprising more than 3000 patients^32^. We did not see a prognostic value of GATA3 gene-expression levels in the present cohort. This contrasts with some other gene-expression studies with microarray data, reviewed by Fang et al, showing that GATA3 is prognostic^33^. Taken together, GATA3 appears as a favorable prognostic factor, but the question of its importance as an independent factor needs further elucidation, considering the choice of cut-off for positivity as well as the influence of breast cancer therapy.

GATA3 is highly correlated with ER and thus a potential predictive marker of benefit from hormonal therapy. Using a breast cancer model with parental and tamoxifen-resistant MCF7 cells, endocrine resistance was associated with downregulation of luminal/epithelial differentiation markers and upregulation of basal/mesenchymal invasive markers^34^. One important factor for the transcriptional landscape was GATA3. Further studies have indicated that GATA3 counteracts EMT. The protein complex GATA3/G9A/MTA3 represses ZEB2, and other genes involved in EMT, leading to suppression of metastasis from human breast cancer cells in mice. In turn, ZEB2 repressed the expression of G9A and MTA3^35^. Moreover, ectopic expression of GATA3 in GATA3-negative triple-negative breast cancer cells led to increased CDH1 expression and decreased expression of some mesenchymal markers^16^. A similar transcriptional change could also be related to mutations of GATA3^36^. Here, the results give support for the experimental findings of an inverse relationship between GATA3 and EMT in a large series of ER-positive tumors. GATA3 expression correlated with high expression of E-cadherin and FOXA1 and low expressions of all five mesenchymal markers investigated.

Besides a prognostic value, one could ask whether GATA3 is a treatment predictive factor. In a minor series of 28 ER-positive cases of breast cancer, lack of GATA3 expression was associated with unresponsiveness to hormonal therapy^37^. Furthermore, GATA3 mRNA expression was associated with longer progression-free survival in patients with ER-positive breast cancer treated with first-line tamoxifen for recurrent disease^38^. To the best of our knowledge, there are no previous reports on the relationship of GATA3 with the efficacy of adjuvant endocrine therapy based on a randomized trial. In the present study, we were able to show that a substantial benefit from tamoxifen was restricted to patients with intermediate/high GATA3 mRNA expression, most evident when focusing on late relapse. We found similar patterns for FOXA1 and E-cadherin, both of which are closely related to GATA3, whereas CDH2 (N-cadherin) and VIM (vimentin) tended to show opposite associations. When the transcriptional landscape of ER is altered upon loss of GATA3, one might speculate that the anti-tumoral effects of tamoxifen is diminished.

One strength with this study is that it is based on a randomized clinical trial and long-term follow-up. The study limitations include that the cohort is confined to postmenopausal patients with lymph-node negative disease, and it is not known if the results related to tamoxifen benefit can be translated to the use of aromatase inhibitors. Another limitation is that we lack data on GATA3 mutations for this cohort. GATA3 is the third most mutated gene in luminal breast cancer. In part, the results of the present study might be applicable to tumors with GATA3 mutation as such mutations affect gene transcription patterns and EMT^36,39,40^.

In conclusion, GATA3 expression is associated with ER-positive breast cancer and particularly with the Luminal A subtype. Diminished expression of GATA3 in ER-positive tumors is associated with changes of gene expression resembling EMT. Both such changes and GATA3 expression itself were related to the efficacy of adjuvant tamoxifen therapy.

## Data Availability

Restrictions apply to the availability of these data according to GDPR. Data were obtained from the STO Trialist Group and are available from the authors with the permission from the STO Trialist Group.
